# Inequalities in referrals to social prescribing from primary care in England: A retrospective observational study

**DOI:** 10.1371/journal.pone.0350842

**Published:** 2026-06-08

**Authors:** Efundem Agboraw, Anna Wilding, Luke Munford, Matt Sutton, Paul Wilson

**Affiliations:** Division of Population Health, Health Services Research and Primary Care, University of Manchester, Manchester, United Kingdom; University of Oxford, UNITED KINGDOM OF GREAT BRITAIN AND NORTHERN IRELAND

## Abstract

**Background:**

Social prescribing is a growing community health intervention and has been associated with improved patient outcomes. However, the evidence around inequalities in referrals shows varying patterns. We aim to examine referrals to social prescribing link workers funded by the Additional Roles Reimbursement Scheme in England.

**Methods:**

We conducted a retrospective observational population-based study, using primary care data from Clinical Practice Research Datalink (CPRD) Aurum from 1st July 2019–31st March 2024. Participants included over 12 million patients aged 16 years or older. We examined the likelihood of offers and subsequent referrals to social prescribing by patient- and area-level characteristics using logistic regression and report odds ratios (ORs).

**Results:**

Since July 2019, approximately 4% of the CPRD population have been offered a referral to social prescribing. 77·7% of those were referred. Patients who are: female, older, living in less deprived areas and have multiple long-term conditions have higher odds of being offered social prescribing (Female OR = 1·35, 95% CI [1·32 to 1·38] p < 0.001). Factors such as region, rurality, and ethnicity do not result in inequalities in offers compared to the general population. Of those offered, we find that those who are female, those from non-white ethnicities (Black, Asian and Mixed), and have multiple long-term conditions had higher odds of accepting offers of referrals (being referred).

**Conclusion:**

Referrals to social prescribing have increased following the national rollout of link workers. However, inequalities in offers and referrals to social prescribing have been identified by patient and area-level factors. Our findings indicate that policies should improve awareness of social prescribing in deprived areas and direct certain patient groups, such as ethnic minorities, males and those older to the benefits of being referred to social prescribing.

## Introduction

Social prescribing (SP) is a growing community health intervention that links patients to non-medical sources of support within their communities. Patients can be referred from primary care [1], local authorities, the voluntary sector, and self-referrals [2], however, the list of referral sources are not exhaustive as they can also be referred from secondary care and schools. Patients then meet with a social prescribing link worker, who facilitates the interventions and identifies their needs and directs them to activities/support such as housing, benefits and employment, arts and culture, exercise, befriending and volunteering [3]. The intervention is aimed at a broad range of populations, but some schemes have specific target populations such as certain disease cohorts [4]. The scheme relies on voluntary and community sector resources to improve self-care and address patients’ health, psychological and social issues [5].

The 2019 NHS Long Term Plan [6] aimed to create more integrated and community-based healthcare to improve health outcomes and tackle health inequalities. From July 2019, groups of general practices could form Primary Care Networks (PCNs), which enabled access to funds from the Additional Roles Reimbursement Scheme (ARRS). Social prescribing link workers were among the first roles for which PCNs could be reimbursed for [6,7]. NHS England aimed to recruit over 1,000 link workers by 2020/21 and refer over 900,000 patients to social prescribing by 2023/24 [8,9]. Later, it was mandated that every PCN should have a social prescribing link worker by 2022 [8]. The ARRS has been successful in the rollout of link workers, with over 3,390 full-time equivalent link workers in post by June 2025 [10].

The ARRS funding schemes use the Carr-Hill Formula to adjust funding allocations for age and sex of PCN populations as well as morbidity and mortality [11]. This in part adjusts for known regional inequalities in long-term conditions (LTCs) [11] and the inverse relationship between healthcare use and need [12]. Despite this, previous research has highlighted that the rollout of social prescribing link workers was negatively related to area-level need, with more deprived areas experiencing lower levels of link worker employment [12] and lower referrals [13, 16].

Past findings indicate that SP interventions can improve patients’ health and well-being, with limited evidence linking them to healthcare utilisation [14]. The evidence around equitable access to social prescribing is emerging, but it focuses predominantly on who is currently being referred, not compared to the general population. One study using referral data from Elemental (social prescribing recording software) showed variations in referrals by demographics and area characteristics across the UK [4]. They found an overrepresentation of women and patients residing in deprived areas and urban areas in England. They found an underrepresentation of older patients, particularly those aged 70 or older. Findings from referral data from 11 general practices in the North West (NW) of England [15] identified similar referral patterns. There was over-representation of women and those residing in the deprived areas; however, they did find a high proportion of older populations being referred. They also examined ethnicity and found higher proportions of non-white minorities being referred, particularly those Black and Mixed. Recent analysis of referred patients to social prescribing using Clinical Practice Research Datalink (CPRD) explored patterns in referrals and decline rates by patient- and area-level characteristics [13, 16]. Similar patterns to the NW study, with a higher proportion in older and non-white ethnicities. However, this study did not look at different ethnic minority groups. They identified patients from less deprived areas with higher referral rates and underrepresentation of rural areas. This was not compared to an unreferred, general population cohort. Finally, a study of referrals amongst 7,283 individuals aged 50 and over using the English Longitudinal Study of Ageing (ELSA) [17] identified that 6.8% received a referral to a community intervention. They found that those who received a referral were from older populations, lower socioeconomic status and had long-term conditions.

Past findings indicate inequalities in referrals, with some studies reporting higher referrals in more deprived areas, while others report the opposite. There is unclear evidence on the age structure and insufficient evidence on morbidity. Furthermore, there is a lack of evidence at a national level on those referred or offered vs. a general population; it focuses on those with an interaction with social prescribing. Our study aims to strengthen the evidence by exploring inequalities and patterns in those offered and referred to social prescribing link workers funded by the ARRS by individual and area-level characteristics. We will assess the intersectionality of these characteristics and compare outcomes with those who do not interact with social prescribing.

## Methods

### Data

We obtained and used electronic healthcare records from the Clinical Practice Research Datalink (CPRD) Aurum (2024 release, accessed on the 15^th^ of January 2025). To obtain the CPRD Aurum dataset, the authors followed the CPRD application process (https://www.cprd.com/research-applications), which entailed submitting a study protocol for approval to the Independent Scientific Advisory Committee (ISAC) to ensure research governance compliance and sign a data license agreement upon approval [18]. The CPRD Aurum dataset covers around 25% of the English population and is broadly representative in terms of age, sex, ethnicity, socioeconomic deprivation and region of England of patients registered in English general practices (GPs) [18]. We link these records to small area-level data (Deprivation and Rurality measures) and CPRD Ethnicity linkages.

**Fig 1:**
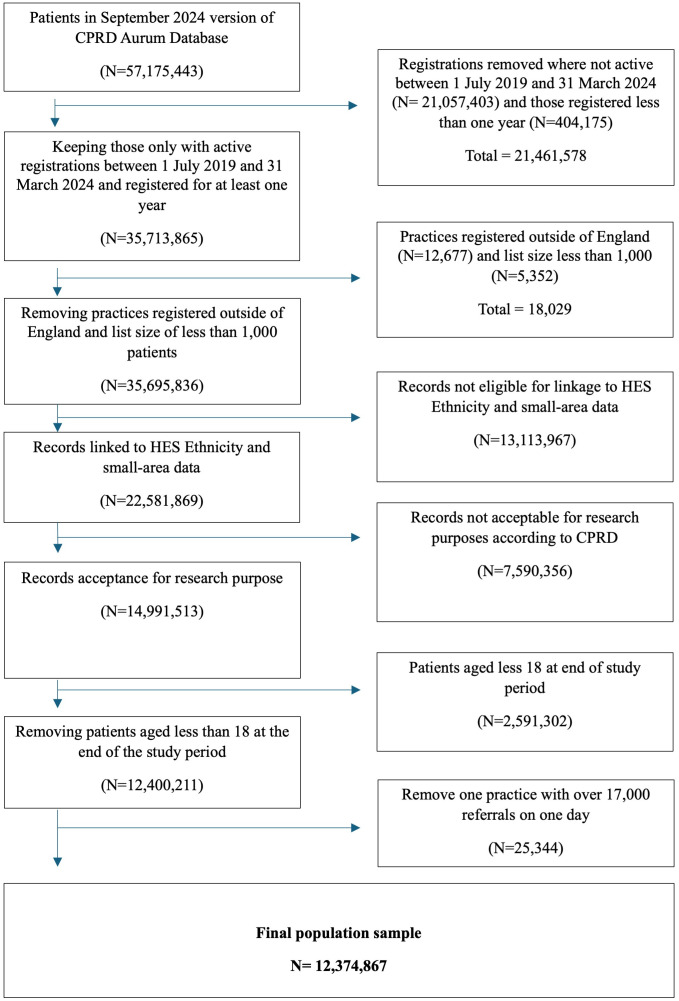
Sample creation of cohort of 12,374,867 patients.

Data extraction was conducted in December 2024. Our inclusion criterion was registered with their general practice for at least one year during the study period (1 July 2019 to 31 March 2024). A dataset of over 57 million patients was extracted. We removed inactive patient registrations and practices registered outside England. From the remaining sample, records that were not eligible for research for HES linkages and safeguarding purposes, following guidance from CPRD [19] were removed (see, Fig 1). We removed an outlier general practice with over 17,000 referrals recorded in one day, due to uncertainty of the validity of this record (Fig 1). This provided a total sample of over 12 million participants.

### Measures

We identified offers and referrals to social prescribing using Systematized Nomenclature of Medicine Clinical Terms (SNOMED) codes extracted from the observation files from CPRD Aurum. Codes are listed in Box 1. These codes were used for identifying recorded referrals to ARRS-funded link workers.

**Box 1. SNOMED Codes for Social Prescribing Interactions.**

- 871691000000100 | Social prescribing offered
- 871711000000103 | Social prescribing declined
- 871731000000106 | Referral to social prescribing service

These SNOMED codes were recommended by the NHS DES contract specifications 2024/25 [20] and Social prescribing reference guide [21] to be used by general practices for the monitoring and collection of ARRS-funded social prescribing service.

### Outcomes

Patients can have multiple mentions of SNOMED codes from Box 1 within their healthcare records. We use any mention of these codes within our study period, 1^st^ July 2019 to 31 March 2024. We split these into two outcomes: offered and referred.

For patients offered social prescribing, it is any mention of the SNOMED codes in Box 1. For referred patients, we used any mention of “Referral to social prescribing service”; this means that if they have offered or declined codes, the referral status overrides those codes.

### Covariates (Inequality variables)

Covariates were chosen to capture a wide range of indivudal- and area-level factors. All variables were measured at the start of the study period, 1 July 2019, except those obtained from dataset linkage (socioeconomic deprivation, ethnicity and practice rurality), which were from September 2024.

For individual-level factors, we include age, sex and ethnicity. The patients’ age was presented in years, which is grouped into eight categories from aged 16–85+ (see Table 1 for groups). For sex, we include a binary variable to indicate whether a patient is female (vs. male). For ethnicity, we include six categories provided by CPRD linkages: Asian, Black, Mixed, White (reference category), Other or unknown. These are derived from the ONS Ethnicity high-level definition [22,23].

**Table 1:** Summary statistics of total population, and offered and referred to social prescribing.

	(1)		(2)		(3)	
	Total population		Offered Social Prescribing		Referred to social prescribing	
Variable	Column %	Number in CPRD	%	Number in CPRD	%	Number in CPRD
Observations	**12,374,867**		**515,892**		**400,848**	
**Age**						
16-19	5·05	624,740	1·26	6,479	1·34	5,381
20-29	17·57	2,174,524	7·84	40,463	7·96	31,900
30-39	18·67	2,310,826	10·85	55,959	10·76	43,137
40-49	15·49	1,916,755	12·39	63,944	12·13	48,632
50-64	21·87	2,706,090	24·58	126,806	24·19	96,984
65-74	10·42	1,290,021	15·48	79,877	14·84	59,470
75-84	7·15	884,344	16·92	87,303	17·29	69,321
85+	3·78	467,557	10·67	55,061	11·48	46,023
**Sex**						
Male	50·08	6,197,595	40·49	208,844	39·10	156,719
Female	49·92	6,176,838	59·51	306,978	60·90	244,102
**Ethnicity**						
Asian	9·8	1,212,403	7·83	40,394	8·38	33,572
Black	4·5	562,348	6·3	32,524	6·51	26,081
Mixed	1·7	210,703	1·41	7,255	1·44	5,759
White	78·66	9,733,975	82·81	427,185	81·98	328,526
Other	0·83	102,143	0·3	1,573	0·29	1,166
Unknown	4·47	553,295	1·35	6,961	1·43	5,744
**Deprivation**						
1 (Most Deprived)	10·95	1,353,722	7·81	40,254	7·9	31,662
2	10·01	1,237,041	8·38	43,223	8·70	34,835
3	9·72	1,201,773	8·31	42,828	8·47	33,954
4	9·61	1,188,479	7·70	39,683	8·16	32,675
5	9·71	1,200,778	8·59	44,277	8·89	35,596
6	9·94	1,229,090	9·39	48,414	9·54	38,721
7	10·58	1,308,199	10·91	56,233	11·07	44,360
8	10·23	1,264,524	11·84	61,070	11·94	47,817
9	10·11	1,250,040	13·03	67,175	12·65	50,665
10 (Least Deprived)	9·14	1,130,489	14·05	72,437	12·69	50,845
**Rurality**						
Urban	88·39	0,937,766	89·01	459,178	88·10	353,165
Rural	11·61	1,437,101	10·99	56,714	11·90	47,683
**Government office regions**						
East Midlands	2·73	338,179	1·32	6,822	1.59	6,022
East	4·3	532,348	4·06	20,968	4.63	18,548
London	21·19	2,622,822	23·8	122,770	23.85	95,600
North East	3·1	383,860	5·04	25,981	4.39	17,598
North West	17·34	2,145,359	21·68	111,820	18.11	72,612
South East	20·06	2,482,148	16·86	86,965	18.53	74,286
South West	11·88	1,470,488	9·65	49,790	10.36	41,537
West Midlands	15·54	1,923,237	15·25	78,685	16.18	64,851
Yorkshire	3·85	476,426	2·34	12,091	2.44	9,794
**Morbidity**						
None	43·23	5,350,091	15·29	78,884	14·07	56,409
1	24·47	3,028,223	18·99	97,951	18·28	73,267
2	15·11	1,869,602	20·92	107,932	21·02	84,250
3	8·51	1,053,504	17·36	895,59	17·78	71,278
4	4·41	545,219	11·90	61,386	12·44	49,874
5+	4·27	528,228	15·54	80,180	16·41	65,770

Note: Deprivation is measured by Index of Multiple Deprivations – Income Domain.

For the area-level factors, we used socio-economic deprivation, rurality, and government regions. Socioeconomic deprivation is at the patient level; we used deciles of the Index of Multiple Deprivation (IMD) 2019 – Income Domain [24]. This is measured at a small area level named Lower Super Output Area with an average population of 1,500 individuals; the top 10% decile is the most deprived, and the bottom 10% is least deprived. Rurality is measured at the general practice level, and we use Rural-Urban classification [25] to create a binary measure if the surgery is practice is located in a rural area. For the government office regions, we used the Office of National Statistics (ONS) measure, which contains nine broad regions listed in Table 1 and measured at the patient level.

To explore the presence of long-term health conditions (LTCs), we extracted 24 of the most prevalent physical and mental health conditions. These were derived from established studies on multimorbidity in England [26]. From this, we create a measure of LTCs of having no long-term conditions, one long-term condition, two long-term conditions, up to five or more long-term conditions.

### Statistical analyses

For descriptive statistics, we present frequencies (number in CPRD) and percentages for each variable.

We use multivariable logistic regression to estimate the probability of being offered social prescribing by individual- and area-level characteristics, and report odds ratios. Conditional on being offered, we estimate the probability of being referred to social prescribing. Given that patients are nested within general practices, we clustered standard errors at the general practice level as we expected their variance to be correlated. We ran three logistic models on outcomes: a) patient characteristics only to explore differences in individual characteristics; b) include area factors to patient characteristics and c) include multimorbidity to explore if health conditions are predictors of referrals. We present coefficient plots for Model C in our main paper, and the results tables are in the Supplementary tables. To assess the intersectionality between characteristics, we estimate interaction models for age and sex, as well as for ethnicity and deprivation. The age-sex interaction was used to explore any additive inequalities which could be present across age and sex, which may ot be picked up in the single categorical analysis. For instance within the sex category, older females may have completely different referral rates than younger females. The interactions between ethnicity and deprivation were conductced to explore any “hidden” inequalities among specific, population groups intersecting with their area of residence which could not be identified in isolation. In these interaction models, we include the other individual and area-level characteristics; these results are in the Supplementary Tables. We conducted statistical analysis in Stata v17.

### Ethics

The ethics for this research (23_002643) was approved by the Clinical Practice Research Datalink (CPRD) Research Data Governance Process for the Medicines and Healthcare products Regulatory Agency (18/09/2023).

## Results

### Descriptive statistics of study population

From 2019–2024, 4% (515,892) of over 12 million patients were offered a referral to social prescribing (column 2, Table 1). Of those offered, 77·7% (400,848) were referred to social prescribing (column 3, Table 1). Patients aged 50 years or older accounted for the majority of those offered and referred (68·3%) (Column 2, Table 1). There were more female patients than males in the offered population (59.5%) and referred (60.9%) populations. For deprivation, there was an over-representation of patients residing in the least deprived areas in both the offered and referred populations, despite an equal distribution in the total population.

Across area-level factors, populations in urban areas remain similar across patient groups. For regions, there is an overrepresentation of patients in the North West. North East and London, and underrepresentation of areas like the South West and South East for those with an interaction with social prescribing. In terms of LTCs, there are higher proportions of patients with multiple long-term conditions in the referred and offered populations than in the total populations.

### Logistic models

#### Offered social prescribing.

**Fig 2:**
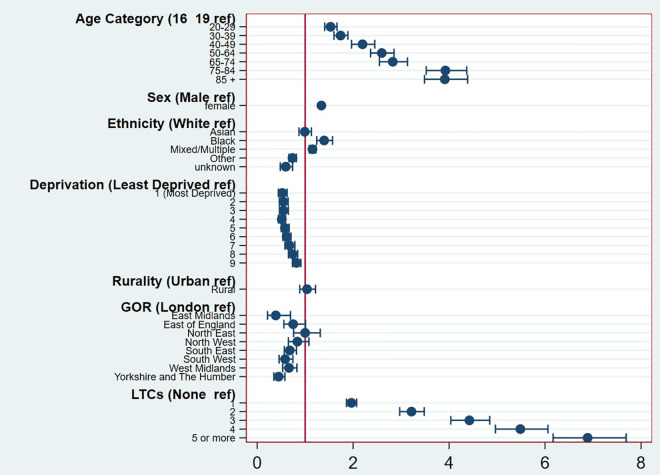
Offered Social Prescribing (Model C).

Fig 2 shows that as age increases, so do the odds ratios of being offered, which is evident in all models (coefficients in S1 Table (Model C)). The odds ratios are consistent between models where we include individual (Model A) and individual- and area-level factors (Model B), however when we include morbidity (Model C) these effects on age decreases.

Females were more likely to be offered social prescribing link worker than males, even after other individual and area factors had been accounted for, including multimorbidity (OR = 1·35, 95% CI [1·32 to 1·38] p < 0.001). These same offered patterns are reflected in the age-sex interactions in S3 Table, where females have systematically higher odds across age categories compared to males. Across ethnicities, in S1 Table, we find Black (OR = 1·39, 95% CI [1·24 to 1·57] p < 0.001) and Mixed ethnicities (OR = 1·15, 95% CI [1·08 to 1·22] p < 0.001) having higher odds than White in being offered social prescribing. While on S3 Table, when interacted with Deprivation, we find no significant differences in the offered social prescribing between those who are from White, Black or Mixed ethnicities; however, we do find that those who are Asian, other or unknown are less likely to be offered.

Patients living in more deprived areas were less likely to be offered social prescribing than those living in the least deprived areas (OR range 0·52–0·82). There were no significant differences in the social prescribing offered across the North West, North East and London regions. However, those in the remaining regions had lower odds of being offered social prescribing.

Similar to age, patients with an increasing number of long-term conditions had increasing odds of being offered social prescribing. For example, patients with five or more long-term conditions had an odds ratio of 3.12 (p < 0.001) compared to patients with no long-term conditions.

### Referred to social prescribing

**Fig 3:**
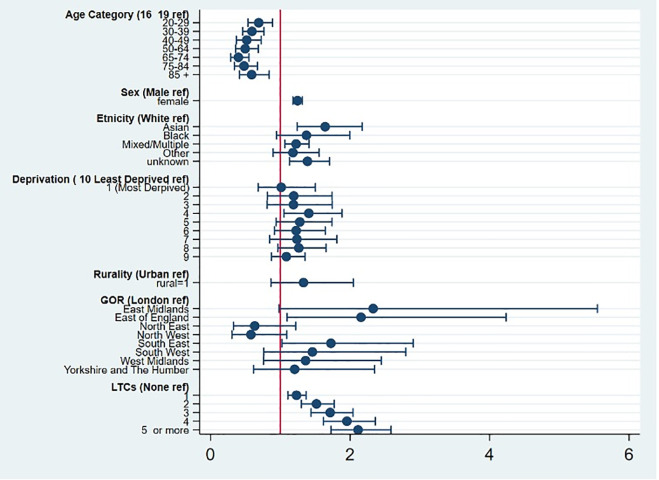
Referred to Social prescribing (Model C).

Fig 3 presents the odds ratios of referrals to social prescribing conditional on being offered. In contrast to the findings in Fig 2, older patients were less likely to be referred when offered (coefficients in S2 Table (Model C)). A similar pattern of odds ratios as in the offered models was present, where is effect was smaller as area (Model B) and morbidity characteristics (Model C) were included. Females were more likely to be referred than males (OR 1.25, 95% CI [1.18 to 3.18], p < 0.001); this magnitude remained consistent across each model. The age-sex interaction showed that female patients were more receptive to referral across all age groups (S3 Table).

For ethnicity, we find that those who are Asian, Mixed or Unknown had higher odds of being referred, conditional on being offered social prescribing. This contrasts with the findings in Fig 2, where, if patients are offered, there is a subsequent higher odds of referral. Looking at the intersectionality with deprivation, we find those who are Asian, black and mixed ethnicities and reside in the middle deciles (4–8) had higher odds of being referred to social prescribing when offered (S3 Table (Model C)).

Area-level factors as a whole were not significant predictors of being referred to social prescribing among those offered. This presents a positive in terms of equity in that their factors, such as rurality, region and deprivation, did not influence outcomes. There were some values, such as the 4^th^ decile of deprivation and those residing in the East of England, that indicated higher odds of referral, but no patterns across these characteristics.

Morbidity was a significant predictor of referrals. Patients with more long-term conditions were more likely to be referred to social prescribing.

## Discussion

### Statement of principal findings

This study analysed primary care data from CPRD Aurum on individual- and area-level characteristics of those offered and/or referred to social prescribing link workers funded through the ARRS to explore the presence of inequalities. Our findings demonstrate that, using recommended SNOMED codes, over 4% of the eligible CPRD population were offered social prescribing. Of those with the offered SNOMED code, 77.7% were recorded with a referred to social prescribing SNOMED code. Our results indicate variations in referral patterns across individual- and area-level factors. The key indicators of being offered social prescribing were those who are female, older populations, those residing in less deprived areas and the presence of long-term conditions. Of those offered, female patients and those with long-term conditions had higher odds of being referred. Factors such as deprivation do not have a significant effect on being referred conditioned on being offered social prescribing. We find opposing effects for age, with older age groups having lower odds of being referred, and ethnicity indicating ethnic minorities are more likely to be referred, when they are offered.

### Strengths and weaknesses

The main strength of this study is the use of nationally representative primary care data from CPRD Aurum with ethnicity and small area linkages data to explore patterns in social prescribing. This allowed for interacting patient and area factors, which account the complexities in social prescribing. The study has some limitations; firstly, our focus is on patients referred from primary care to the additional roles reimbursement scheme service. Referrals to social prescribing services via other routes, such as social care [13, 16], are not included, and as such our findings may not represent the full picture of who accesses social prescribing. Secondly, we only look at referrals to social prescribing. Within electronic healthcare records, uptake is not recorded. However, past evidence using social prescribing platform data found 90% of referrals led to patients seeing a link worker, but only 38% of those received an intervention [4]. Our findings should be interpreted with caution. Thirdly, the reasons for social prescribing referrals can include wider social determinants, such as financial indebtedness or employment issues. This goes beyond the individual- and area-level characteristics we include in our models. Some of these reasons have been identified in smaller survey data analyses of referrals [17]; this is not feasible in our study. Finally, exploring patterns of offers and referrals to social prescribing using SNOMED codes relies on reliable coding from healthcare professionals. We may have misreporting of offers of social prescribing as there are incentives relating to “referral to social prescribing service”, not the other codes.

### Findings in context with existing literature

A recent paper by Bu, F et al (2025) [16] also measured the national rollout of social prescribing using CPRD. The findings were similar to this study in terms of referrals and declines by patient demographics and deprivation. While Bu et al. compared referral trends annually and provided an annual multilevel analysis, this study provided an aggregate analysis of the rollout of social prescribing by patient and area-level characteristics. We also provide additional evidence in two main ways. First, we consider and additional characteristic, including long term conditions and individual ethnicity categories. Secondly, we analyse offers of social prescribing in comparison to the total population.

Other studies exploring patterns in social prescribing focus more on the referral acceptance and decline rates, and less on the population being offered referrals in the first instance. However, the patterns of offer of referrals in our study match studies exploring referral rates in social prescribing [3,15,16,27] and patterns of acceptance in our study match studies that explored both referred and declined referrals [15,16]. In line with our findings, most studies show age as a predictor of referrals, with older people being more likely to be referred [15–20] and these same older patients having lower odds of accepting referrals when offered [15,19]. Current literature also shows sex as a consistent predictor of social prescribing referrals, where female patients were likely to be referred. This is illustrated in recent studies done in the North West of England [15], with databases like Elemental [3], ELSA [17] and CPRD and social prescribing reports like the Rotterdam Social prescribing report (2014) [28] and the NASP review on SP studies [29]. On the other hand, there have been concerns on the underrepresentation of ethnic minorities and other ethnically diverse population groups having lower access and engagement to social prescribing [13,15]. However, findings in our study suggest that ethnic minorities significantly accept referrals when offered. Furthermore, in line with our study, morbidity has been identified as a major cause of social prescribing referrals [30]. One of the aims of the NHS social prescribing scheme is improved health outcomes through management of LTCs, hence recommended that social prescribing be targeted towards patients with multimorbidity and chronic illnesses [6, 31, 32].

Social prescribing is aimed at reducing health inequalities; however, concerns have been raised about its potential to exacerbate existing inequalities by helping the less disadvantaged population more [33]. This is evident in the area-level disparities identified in our findings, where offered referrals were disproportionately found in the least deprived areas. These findings corroborate studies on patterns of social prescribing referrals [3,13–16,27]. From our models on referrals given, being offered social prescribing demonstrates that area-level factors do not influence referrals. This has the potential to improve health inequalities, as long as access to social prescribing improves, there should be no inequities in terms of these factors.

### Conclusion (Implications for policy and research)

We find inequalities in access to social prescribing driven by patient-level factors such as age and sex, and by area-level factors such as deprivation. There needs to be tailored approaches to improve offers to social prescribing, particularly for those residing in the most deprived areas. In terms of equity, we do not find significant differences between those referred and offered in terms of deprivation. This indicates access is an issue rather than uptake. However, for factors such as sex, females have systematically higher offers and referrals, and tailored approaches to understand why males are underrepresented in social prescribing. The NHS Social Prescribing scheme is directed towards those with long-term conditions. We find a higher proportion of patients being offered and referred, indicating that the policy has been successfully delivered in that regard.

To ensure the sustainability of social prescribing, there is the need for further research into the reasons for the existing inequalities in social prescribing, to develop more contextual, tailored and strategic referrals systems that ensures improved and equitable referrals and engagement to social prescribing. An approach to do this would be to improve the integration of data from electronic healthcare records and social prescribing software. This means a better understanding of who is referred, the reasons for the referral, and whether they received an intervention. This would allow for a more in-depth analysis of individual- and area-level factors and monitor the use of SNOMED codes to assess coding reliability in healthcare records.

## Supporting information

S1 Table Odd ratios of offers to social prescribing with 95% confidence interval. (DOCX)

S2 Table Odd ratios of referrals to social prescribing conditional on being offered with 95% confidence interval. (DOCX)

S3 Table Logistic models with interaction between age and sex and ethnicity and Deprivation. (DOCX)
