# Supplementary material for: Inequalities in referrals to social prescribing from primary care in England: A retrospective observational study
Source: PLoS One. 2026 Jun 8;21(6):e0350842. doi: 10.1371/journal.pone.0350842 (PMC13245770; doi:10.1371/journal.pone.0350842)
Supplement: S1 Table — (DOCX) [file pone.0350842.s001.docx]

**S1 Table.** **Odd ratios of offers to social prescribing with 95% confidence interval**

| **Model** | **A** | **B** | **C** |
| --- | --- | --- | --- |
|  | **Individual** | **Individual + Area** | **Individual + Area + Morbidity** |
| **Age** |  |  |  |
| 16-19 | Ref | Ref | Ref |
| 20-29 | 1·840*** | 1·869*** | 1·529*** |
|  | (1·679 to 2·015) | (1·719 to 2·032) | (1·406 to 1·663) |
| 30-39 | 2·388*** | 2·301*** | 1·739*** |
|  | (2·203 to 2·588) | (2·129 to 2·485) | (1·600 to 1.890) |
| 40-49 | 3·308*** | 3·300*** | 2·195*** |
|  | (2·970 to 3·684) | (2·974 to 3·386) | (1.966 to 2·450) |
| 50-64 | 4·726*** | 4·832*** | 2·596*** |
|  | (4·299 to 5·194) | (4·399 to 5·306) | (2·363 to 2·852) |
| 65 - 74 | 6·370*** | 6·703*** | 2·825*** |
|  | (5·706 to 7·111) | (6·021 to 7·461) | (2·548 to 3·132) |
| 75 - 84 | 10·465*** | 11·269*** | 4·589*** |
|  | (9·243 to 11·849) | (10·009 to 12·687) | (4·411 to 5·122) |
| 85+ | 12·428*** | 13·380*** | 4·546*** |
|  | (10·858 to 14·223) | (11·769 to 15·211) | (4·044 to 5·111) |
| **Sex** |  |  |  |
| Male | Ref | Ref | Ref |
| Female | 1·417*** | 1·424*** | 1·353*** |
|  | (1·484 to 1·450) | (1·392 to 1·456) | (1·324 to 1·383) |
| **Ethnicity** |  |  |  |
| White | Ref | Ref | Ref |
| Asian | 1·034 | 0·886 | 0·992 |
|  | (0·906 to 1·179) | (0·779 to 1·005) | (0·871 to 1·130) |
| Black | 1·721*** | 1·250*** | 1·396*** |
|  | (1·513 to 1·957) | (1·115 to 1·400) | (1·241 to 1·570) |
| Mixed | 1·196*** | 1·044 | 1·152*** |
|  | (1·110 to 1·289) | (0·984 to 1·107) | (1·086 to 1·223) |
| Other | 0·565*** | 0·483*** | 0·641*** |
|  | (0·507 to 0·6629) | (0·437 to 0·533) | (0·579 to 0·710) |
| Unknown | 0·388*** | 0·402*** | 0·533*** |
|  | (0·310 to 0·485) | (0·321 to 0·502) | (0·429 to 0·662) |
| **Deprivation Deciles** |  |  |  |
| 1 |  | 0·435*** | 0·519*** |
|  |  | (0·368 to 0·514) | (0·437 to 0·617) |
| 2 |  | 0·468*** | 0·542*** |
|  |  | (0·398 to 0·549) | (0·459 to 0·640) |
| 3 |  | 0·478*** | 0·546*** |
|  |  | (0·406 to 0·562) | (0·462 to 0·645) |
| 4 |  | 0·452*** | 0·509*** |
|  |  | (0·392 to 0·520) | (0·440 to 0·589) |
| 5 |  | 0·517*** | 0·574*** |
|  |  | (0·450 to 0·592) | (0·499 to 0·662) |
| 6 |  | 0·560*** | 0·611*** |
|  |  | (0·490 to 0·638) | (0·532 to 0·699) |
| 7 |  | 0·625*** | 0·670*** |
|  |  | (0·538 to 0·726) | (0·574 to 0·781) |
| 8 |  | 0·703*** | 0·739*** |
|  |  | (0·620 to 0·797) | (0·649 to 0·840) |
| 9 |  | 0·795*** | 0·817*** |
|  |  | (0·716 to 0·882) | (0·734 to 0·909) |
| 10 |  | Ref | Ref |
|  |  |  |  |
| Rural |  | 1·019 | 1·043 |
|  |  | (0·869 to 1·194) | (0·890 to 1·221) |
| **Government office regions** |  |  |  |
| London |  | Ref | Ref |
| East Midlands |  | 0·431*** | 0·393*** |
|  |  | (0·242 to 0·768) | (0·219 to 0·702) |
| East |  | 0·801 | 0·763 |
|  |  | (0·598 to 1·075) | (0·566 to 1·342) |
| North East |  | 1·162 | 1·02 |
|  |  | (0·886 to 1·524) | (0·775 to 1·342) |
| North West |  | 0·945 | 0·852 |
|  |  | (0·739 to 1·208) | (0·661 to 1·096) |
| South East |  | 0·735*** | 0·690*** |
|  |  | (0·614 to 0·882) | (0·574 to 0·892) |
| South West |  | 0·640*** | 0·594*** |
|  |  | (0·503 to 0·815) | (0·465 to 0·758) |
| West Midlands |  | 0·738*** | 0·675*** |
|  |  | (0·593 to 0·918) | (0·541 to 0·843) |
| Yorkshire & Humber |  | 0·494*** | 0·456*** |
|  |  | (0·383 to 0·636) | (0·354 to 0·588) |
| **LTCs** |  |  |  |
| None |  |  | Ref |
| 1 |  |  | 1·964*** |
|  |  |  | (1·861 to 2·072) |
| 2 |  |  | 3·216*** |
|  |  |  | (2·970 to 3·481) |
| 3 |  |  | 4·422*** |
|  |  |  | (4·036 to 4·486) |
| 4 |  |  | 5·485*** |
|  |  |  | (4·965 to 6·059) |
| 5+ |  |  | 6·886*** |
|  |  |  | (6·167 to 7·690) |
| N | 12,363,699 | 12,363,699 | 12,363,699 |
| ***Note:*** *95% Confidence Intervals based on clustered standard errors at general practice level (N=1464) level in parentheses. * p<0.05, ** p<0.01, *** p<0.001* | | | |
