# Supplementary material for: Inequalities in referrals to social prescribing from primary care in England: A retrospective observational study
Source: PLoS One. 2026 Jun 8;21(6):e0350842. doi: 10.1371/journal.pone.0350842 (PMC13245770; doi:10.1371/journal.pone.0350842)
Supplement: S2 Table — (DOCX) [file pone.0350842.s002.docx]

**S2 Table.** **Odd ratios of referrals to social prescribing conditional on being offered with 95% confidence interval**

| **Model** | **A** | **B** | **C** |
| --- | --- | --- | --- |
|  | **Individual** | **Individual + Area** | **Individual + Area + Morbidity** |
| **Age** |  |  |  |
| 16-19 | Ref | Ref | Ref |
| 20-29 | 0·755* | 0·761* | 0·704** |
|  | (0·587 to 0·971) | (0·595 to 0·972) | (0·550 to 0·902) |
| 30-39 | 0·674** | 0·678** | 0·607*** |
|  | (0·517 to 0·879) | (0·531 to 0·866) | (0·473 to 0·778) |
| 40-49 | 0·633* | 0·621** | 0·534*** |
|  | (0·445 to 0·901) | (0·442 to 0·873) | (0·382 to 0·747) |
| 50-64 | 0·672* | 0·646* | 0·516*** |
|  | (0·475 to 0·950) | (0·459 to 0·909) | (0·373 to 0·714) |
| 65 - 74 | 0·613* | 0·561** | 0·416*** |
|  | (0·420 to 0·895) | (0·394 to 0·800) | (0·300 to 0·577) |
| 75 - 84 | 0·815 | 0·712 | 0·501*** |
|  | (0·537 to 1·238) | (0·487 to 1·039) | (0·357 to 0·703) |
| 85+ | 1·073 | 0·91 | 0·615** |
|  | (0·691 to 1·664) | (0·609 to 1·359) | (0·430 to 0·878) |
| **Sex** |  |  |  |
| Male | Ref | Ref | Ref |
| Female | 1·284*** | 1·265*** | 1·249*** |
|  | (1·215 to 1·358) | (1·196 to 1·337) | (1·184 to 1·318) |
| **Ethnicity** |  |  |  |
| White | Ref | Ref | Ref |
| Asian | 1·552 | 1·568** | 1·630*** |
|  | (1·213 to 1·985) | (1·187 to 2·071) | (1·233 to 2·153) |
| Black | 1·265 | 1·306 | 1·369 |
|  | (0·850 to 1·881) | (0·900 to 1·896) | (0·944 to 1·987) |
| Mixed | 1·196 | 1·188* | 1·222** |
|  | (1·044 to 1·402) | (1·032 to 1·312) | (1·061 to 1·340) |
| Other | 0·931 | 0·996 | 1·134 |
|  | (0·690 to 1·257) | (0·765 to 1·297) | (0·864 to 1·489) |
| Unknown | 1·455** | 1·276* | 1·367** |
|  | (1·119 to 1·891) | (1·040 to 1·566) | (1·113 to 1·679) |
| **Deprivation Deciles** |  |  |  |
| 1 |  | 0·946 | 1·011 |
|  |  | (0·637 to 1·406) | (0·681 to 1·500) |
| 2 |  | 1·125 | 1·19 |
|  |  | (0·768 to 1·649) | (0·814 to 1·739) |
| 3 |  | 1·132 | 1·188 |
|  |  | (0·768 to 1·668) | (0·809 to 1·744) |
| 4 |  | 1·364* | 1·409* |
|  |  | (1·021 to 1·821) | (1·054 to 1·883) |
| 5 |  | 1·239 | 1·28 |
|  |  | (0·908 to 1·690) | (0·940 to 1·741) |
| 6 |  | 1·198 | 1·227 |
|  |  | (0·892 to 1·610) | (0·916 to 1·644) |
| 7 |  | 1·218 | 1·239 |
|  |  | (0·829 to 1·790) | (0·847 to 1·813) |
| 8 |  | 1·256 | 1·266 |
|  |  | (0·957 to 1·649) | (0·967 to 1·658) |
| 9 |  | 1·09 | 1·089 |
|  |  | (0·873 to 1·361) | (0·874 to 1·357) |
| 10 |  | Ref | Ref |
|  |  |  |  |
| Rural |  | 1·33 | 1·335 |
|  |  | (0·862 to 2·051) | (0·868 to 2·053) |
| **Government office regions** |  |  |  |
| London |  | Ref | Ref |
| East Midlands |  | 2·344* | 2·331 |
|  |  | (1·013 to 5·423) | (0·980 to 5·543) |
| East |  | 2·199* | 2·163* |
|  |  | (1·123 to 4·306) | (1·101 to 4·251) |
| North East |  | 0·68 | 0·638 |
|  |  | (0·350 to 1·322) | (0·330 to 1·233) |
| North West |  | 0·592 | 0·579 |
|  |  | (0·311 to 1·127) | (0·306 to 1·096) |
| South East |  | 1·770* | 1·732* |
|  |  | (1·045 to 2·999) | (1·027 to 2·920) |
| South West |  | 1·513 | 1·465 |
|  |  | (0·786 to 2·914) | (0·764 to 2·813) |
| West midlands |  | 1·409 | 1·37 |
|  |  | (0·777 to 2·557) | (0·761 to 2·465) |
| Yorkshire & Humber |  | 1·269 | 1·211 |
|  |  | (0·649 to 2·480) | (0·620 to 2·369) |
| **LTCs** |  |  |  |
| None |  |  | Ref |
| 1 |  |  | 1·231*** |
|  |  |  | (1·107 to 1·368) |
| 2 |  |  | 1·517*** |
|  |  |  | (1·300 to 1·772) |
| 3 |  |  | 1·714*** |
|  |  |  | (1·440 to 2·041) |
| 4 |  |  | 1·954*** |
|  |  |  | (1·617 to 2·360) |
| 5+ |  |  | 2·114*** |
|  |  |  | (1·727 to 2·587) |
| N | 515,892 | 515,892 | 515,892 |

***Note:*** *95% Confidence Intervals based on clustered standard errors at general practice level (N=1405) level in parentheses. * p<0.05, ** p<0.01, *** p<0.001*
