# Supplementary material for: Inequalities in referrals to social prescribing from primary care in England: A retrospective observational study
Source: PLoS One. 2026 Jun 8;21(6):e0350842. doi: 10.1371/journal.pone.0350842 (PMC13245770; doi:10.1371/journal.pone.0350842)
Supplement: S3 Table — (DOCX) [file pone.0350842.s003.docx]

**S3 Table.** **Logistic models with interaction between age and sex and ethnicity and Deprivation**

|  | Offered | Referred |
| --- | --- | --- |
| **Age and Sex** |  |  |
| **16-19 Male** | **Ref** | **Ref** |
| 16-19 Female | 1·585*** | 0·956 |
|  | (1·375 to 1·826) | (0·808 to 1·132) |
| 20-29 Male | 2·309*** | 0·640** |
|  | (1·751 to 3·046) | (0·482 to 0·850) |
| 20-29 Female | 2·916*** | 0·699* |
|  | (2·188 to 3·887) | (0·522 to 0·936) |
| 30-39 Male | 2·855*** | 0·523*** |
|  | (2·125 to 3·836) | (0·387 to 0·708) |
| 30-39 Female | 3·749*** | 0·621*** |
|  | (2·847 to 4·936) | (0·472 to 0·818) |
| 40-49 Male | 3·989*** | 0·458*** |
|  | (2·637 to 6·033) | (0·309 to 0·680) |
| 40-49 Female | 5·241*** | 0·543** |
|  | (3·539 to 7·762) | (0·376 to 0·784) |
| 50-64 Male | 5·344*** | 0·427*** |
|  | (3·702 to 7·714) | (0·295 to 0·616) |
| 50-64 Female | 5·948*** | 0·529*** |
|  | (4·161 to 8·504) | (0·372 to 0·754) |
| 65-74 Male | 7·264*** | 0·326*** |
|  | (4·961 to 10·638) | (0·225 to 0·472) |
| 65-74 Female | 7·240*** | 0·445*** |
|  | (4·971 to 10·545) | (0·310 to 0·639) |
| 75-84 Male | 8·608*** | 0·390*** |
|  | (5·789 to 12·798) | (0·267 to 0·568) |
| 75-84 Female | 8·497*** | 0·536** |
|  | (5·714 to 12·635) | (0·368 to 0·781) |
| 85+ Male | 7·790*** | 0·524** |
|  | (5·180 to 11·715) | (0·354 to 0·776) |
| 85+ Female | 7·145*** | 0·615* |
|  | (4·744 to 10·760) | (0·416 to 0·911) |
| **Ethnicity and deprivation** |  |  |
| **White Deprivation 10 (least deprived)** | **Ref** | **Ref** |
| Asian Deprivation 1 (most deprived) | 0·298*** | 1·147 |
|  | (0·173 to 0·512) | (0·701 to 1·876) |
| Asian Deprivation 2 | 0·301*** | 1·692* |
|  | (0·187 to 0·486) | (1·042 to 2·746) |
| Asian Deprivation 3 | 0·309*** | 1·811* |
|  | (0·189 to 0·506) | (1·129 to 2·904) |
| Asian Deprivation 4 | 0·220*** | 2·386*** |
|  | (0·141 to 0·344) | (1·536 to 3·705) |
| Asian Deprivation 5 | 0·295*** | 2·111** |
|  | (0·182 to 0·480) | (1·300 to 3·427) |
| Asian Deprivation 6 | 0·288*** | 2·352*** |
|  | (0·184 to 0·451) | (1·534 to 3·605) |
| Asian Deprivation 7 | 0·323*** | 2·358*** |
|  | (0·227 to 0·459) | (1·687 to 3·297) |
| Asian Deprivation 8 | 0·360*** | 2·236*** |
|  | (0·236 to 0·549) | (1·497 to 3·340) |
| Asian Deprivation 9 | 0·609* | 1·686* |
|  | (0·380 to 0·977) | (1·125 to 2·525) |
| Asian Deprivation 10 (least deprived) | 0·679 | 1·505 |
|  | (0·401 to 1·149) | (0·977 to 2·316) |
| Black Deprivation 1 (most deprived) | 0·450** | 1·233 |
|  | (0·254 to 0·797) | (0·714 to 2·129) |
| Black Deprivation 2 | 0·353*** | 2·154** |
|  | (0·203 to 0·613) | (1·250 to 3·712) |
| Black Deprivation 3 | 0·476** | 1·586 |
|  | (0·285 to 0·795) | (0·963 to 2·612) |
| Black Deprivation 4 | 0·459*** | 1·917** |
|  | (0·304 to 0·694) | (1·256 to 2·926) |
| Black Deprivation 5 | 0·423*** | 2·305*** |
|  | (0·289 to 0·620) | (1·560 to 3·404) |
| Black Deprivation 6 | 0·496** | 2·193** |
|  | (0·311 to 0·789) | (1·365 to 3·522) |
| Black Deprivation 7 | 0·686* | 1·788** |
|  | (0·481 to 0·979) | (1·175 to 2·722) |
| Black Deprivation 8 | 0·635 | 1·897* |
|  | (0·377 to 1·067) | (1·106 to 3·253) |
| Black Deprivation 9 | 0·9 | 1·273 |
|  | (0·503 to 1·609) | (0·723 to 2·241) |
| Black Deprivation 10 (least deprived) | 0·851 | 1·292 |
|  | (0·561 to 1·289) | (0·873 to 1·912) |
| Mixed Deprivation 1 (most deprived) | 0·371** | 1·177 |
|  | (0·199 to 0·692) | (0·625 to 2·216) |
| Mixed Deprivation 2 | 0·447** | 1·358 |
|  | (0·260 to 0·766) | (0·792 to 2·331) |
| Mixed Deprivation 3 | 0·429** | 1·385 |
|  | (0·249 to 0·739) | (0·822 to 2·333) |
| Mixed Deprivation 4 | 0·351*** | 1·734* |
|  | (0·210 to 0·586) | (1·072 to 2·806) |
| Mixed Deprivation 5 | 0·309*** | 2·166*** |
|  | (0·198 to 0·481) | (1·400 to 3·351) |
| Mixed Deprivation 6 | 0·413*** | 1·826** |
|  | (0·264 to 0·645) | (1·203 to 2·771) |
| Mixed Deprivation 7 | 0·684 | 1·2 |
|  | (0·385 to 1·215) | (0·708 to 2·034) |
| Mixed Deprivation 8 | 0·528** | 1·774** |
|  | (0·359 to 0·777) | (1·237 to 2·543) |
| Mixed Deprivation 9 | 0·775 | 1·236 |
|  | (0·519 to 1·157) | (0·844 to 1·810) |
| Mixed Deprivation 10 (least deprived) | 0·985 | 1·2 |
|  | (0·710 to 1·364) | (0·892 to 1·616) |
| White Deprivation 1 (most deprived) | 0·482*** | 1·026 |
|  | (0·315 to 0·738) | (0·683 to 1·540) |
| White Deprivation 2 | 0·440*** | 1·194 |
|  | (0·286 to 0·677) | (0·799 to 1·784) |
| White Deprivation 3 | 0·467*** | 1·183 |
|  | (0·301 to 0·725) | (0·789 to 1·774) |
| White Deprivation 4 | 0·374*** | 1·398* |
|  | (0·269 to 0·521) | (1·019 to 1·918) |
| White Deprivation 5 | 0·474*** | 1·25 |
|  | (0·336 to 0·667) | (0·896 to 1·745) |
| White Deprivation 6 | 0·540*** | 1·177 |
|  | (0·391 to 0·746) | (0·864 to 1·604) |
| White Deprivation 7 | 0·556** | 1·212 |
|  | (0·358 to 0·864) | (0·801 to 1·833) |
| White Deprivation 8 | 0·617** | 1·228 |
|  | (0·446 to 0·855) | (0·911 to 1·656) |
| White Deprivation 9 | 0·700** | 1·113 |
|  | (0·547 to 0·896) | (0·883 to 1·405) |
| Other Deprivation 1 (most deprived) | 0·272* | 0·844 |
|  | (0·100 to 0·741) | (0·309 to 2·304) |
| Other Deprivation 2 | 0·223** | 1·572 |
|  | (0·076 to 0·656) | (0·487 to 5·074) |
| Other Deprivation 3 | 0·191*** | 2·083 |
|  | (0·091 to 0·403) | (0·916 to 4·733) |
| Other Deprivation 4 | 0·285*** | 1·219 |
|  | (0·149 to 0·543) | (0·615 to 2·415) |
| Other Deprivation 5 | 0·270*** | 1·459 |
|  | (0·140 to 0·522) | (0·707 to 3·009) |
| Other Deprivation 6 | 0·225*** | 1·496 |
|  | (0·115 to 0·441) | (0·721 to 3·101) |
| Other Deprivation 7 | 0·379** | 1·281 |
|  | (0·203 to 0·710) | (0·686 to 2·392) |
| Other Deprivation 8 | 0·407*** | 1·492 |
|  | (0·256 to 0·647) | (0·928 to 2·400) |
| Other Deprivation 9 | 0·538* | 1·248 |
|  | (0·326 to 0·888) | (0·770 to 2·020) |
| Other Deprivation 10 (least deprived) | 0·734 | 1·321 |
|  | (0·412 to 1·309) | (0·769 to 2·271) |
| Unknown Deprivation 1 (most deprived) | 0·216*** | 0·888 |
|  | (0·104 to 0·452) | (0·439 to 1·797) |
| Unknown Deprivation 2 | 0·299*** | 0·961 |
|  | (0·159 to 0·561) | (0·509 to 1·815) |
| Unknown Deprivation 3 | 0·237*** | 1·412 |
|  | (0·136 to 0·413) | (0·860 to 2·317) |
| Unknown Deprivation 4 | 0·171*** | 1·668* |
|  | (0·103 to 0·284) | (1·052 to 2·646) |
| Unknown Deprivation 5 | 0·177*** | 1·950** |
|  | (0·106 to 0·296) | (1·259 to 3·019) |
| Unknown Deprivation 6 | 0·170*** | 2·163*** |
|  | (0·110 to 0·263) | (1·406 to 3·328) |
| Unknown Deprivation 7 | 0·252*** | 1·637* |
|  | (0·158 to 0·401) | (1·009 to 2·655) |
| Unknown Deprivation 8 | 0·213*** | 1·919** |
|  | (0·134 to 0·338) | (1·271 to 2·896) |
| Unknown Deprivation 9 | 0·262*** | 1·909** |
|  | (0·158 to 0·434) | (1·290 to 2·826) |
| Unknown Deprivation 10 (least deprived) | 0·258*** | 2·196*** |
|  | (0·158 to 0·422) | (1·450 to 3·326) |
| **Urban** | **Ref** | **Ref** |
| Rural | 0·798 | 1·337 |
|  | (0·516 to 1·234) | (0·870 to 2·054) |
| **London** | **Ref** | **Ref** |
| East Midlands | 0·226*** | 2·349 |
|  | (0·113 to 0·452) | (0·987 to 5·589) |
| East | 0·410** | 2·181* |
|  | (0·215 to 0·781) | (1·111 to 4·281) |
| North East | 1·407 | 0·636 |
|  | (0·715 to 2·767) | (0·330 to 1·227) |
| North West | 1·315 | 0·581 |
|  | (0·686 to 2·521) | (0·307 to 1·099) |
| South East | 0·459** | 1·739* |
|  | (0·276 to 0·763) | (1·032 to 2·933) |
| South West | 0·454* | 1·472 |
|  | (0·239 to 0·862) | (0·767 to 2·825) |
| West midlands | 0·539 | 1·376 |
|  | (0·290 to 1·001) | (0·764 to 2·477) |
| Yorkshire & Humber | 0·400** | 1·21 |
|  | (0·211 to 0·760) | (0·620 to 2·362) |
| **None** | **Ref** | **Ref** |
| 1 | 1·588*** | 1·231*** |
|  | (1·451 to 1·739) | (1·109 to 1·367) |
| 2 | 2·104*** | 1·519*** |
|  | (1·814 to 2·440) | (1·304 to 1·770) |
| 3 | 2·538*** | 1·716*** |
|  | (2·128 to 3·027) | (1·444 to 2·039) |
| 4 | 2·756*** | 1·955*** |
|  | (2·260 to 3·361) | (1·621 to 2·358) |
| 5 + | 3·124*** | 2·115*** |
|  | (2·495 to 3·911) | (1·730 to 2·586) |
| **N** | **12,363,699** | **515,564** |

***Note:*** *95% Confidence Intervals based on clustered standard errors at general practice level (N=1464 for offered and N= 1405 for referred) level in parentheses. * p<0.05, ** p<0.01, *** p<0.001*
